# Kinematic, arm-stroke efficiency, coordination, and energetic parameters of the 400-m front-crawl test: A meta-analysis

**DOI:** 10.3389/fspor.2023.977739

**Published:** 2023-01-26

**Authors:** Ricardo de Assis Correia, Wellington Gomes Feitosa, Flávio Antônio de Souza Castro

**Affiliations:** ^1^ Aquatic Sports Research Group, School of Physical Education, Physiotherapy and Dance, Federal University of the Rio Grande do Sul, Porto Alegre, Brazil; ^2^ Faculty of Physical Education, Ceará State University, Fortaleza, Brazil

**Keywords:** swimming, biomechanics, systematic review, performance, middle-distance

## Abstract

Several studies have investigated biomechanical and energetic parameters in competitive swimming. Among these studies, it is possible to identify the 400-m front crawl as a useful test to assess these parameters. The present study provided a meta-analysis assessing representative variables for the kinematic, arm-stroke efficiency, coordination, and energetic parameters of the 400-m front crawl test. *PubMed*, *Embase*, *Web of Science*, and *SPORTDiscus* were the databases used to select the studies published between January 1970 and December 2022. Forty studies (*n* = 651 swimmers) were selected according to the eligibility and inclusion criteria. The variables chosen to represent each parameter were: clean swim speed (kinematics); index of coordination (coordination); arm-stroke efficiency (efficiency); and oxygen consumption (energetic). Swimming speed was moderate (1.34 m s^−1^) compared to the world's records performers. Thus, this speed contributed for the swimmers in remaining at high efficiency (35%), imposing a capture coordination model (index of coordination: −11%) with high oxygen consumption (58.8 ml·kg^−1^ min^−1^). High heterogeneity (>75%) was found among the outcome parameters in the studies. The different average speeds that represented the kinematic parameters seem to be the most responsible and influential in the arm-stroke efficiency, coordination, and energetic parameters for high 400-m freestyle (front crawl) performance. This meta-analysis can help researchers, coaches, and swimmers improving competitive performance, and developing further research in the sports sciences area, specifically in the swimming.

## Introduction

The swimming performance is influenced by kinematic, arm-stroke efficiency, coordination, and energetic parameters (1) and those have been investigated in scientific research (2–5). The synthesis of scientific research results on such parameters can be useful for coaches and researchers to monitor, improve performance, and develop future research. The 400-m, even test (front crawl) or competitive event (freestyle), is a middle-distance swimming distance (6). The descriptive and quantitative summary of 400-m front crawl test specific performance parameters (such as kinematic, arm-stroke efficiency, coordination, and energetic) seems to have not been explored yet.

The 400-m test and competitive event has a prevalence of a parabolic pacing patterns or a fast-even of swimming speed (7), and is performed under the severe intensity domain (8). The mean swimming speed is the product of the mean stroke rate (SR) and mean stroke length (SL), without the effect of the wall push-off, turns and start effects (2). In a middle-distance event, SR and SL can vary (9) according to gender (male and female), categories (age-groups or adults), training experience, and levels of swimming technical skills. The 400-m front-crawl test is widely used in different competitive levels and the elite swimmers in this event can reach speeds ranging from 1.58 to 1.76 m s^−1^ (10). In addition, to reach such speeds, a refined technique can help the swimmer in reduce hydrodynamic drag and generating propulsion. The relation between the swimmer's applied force and effective propulsion is the arm-stroke efficiency (ηp). There are different possibilities to estimate ηp (11) as the percentage of the force generated by the swimmers that is actually propulsive. A model previously proposed (3) considers the stroke cycle as a paddle wheel producing force to propel the swimmer forward. With this method, the *ηp* values for the 400-m front crawl were around 30%–40% (12–14). Three dimensional (3D) *ηp* analysis, which considers both the 3D centre of mass speed and 3D hand speed, can be applied too (11). The coordination parameters can be represented, in the front crawl, by the index of coordination (IdC) (4). The IdC characterizes what coordination model the swimmer adopts, i.e., the IdC negative (catch-up model), null (opposition model), and positive (superposition model). In the 400-m front crawl test, studies (14–16) indicated that swimmers adopt a non-propulsive interval between the actions of the arm-stroke, the catch-up model. Expert swimmers in this distance present an IdC from −20% to −10% (17, 18).

However, performing any swimming stroke, under any coordination model, to reach a certain swimming speed requires an amount of metabolic energy. In this way, the bioenergetics profile can be determined by VO_2_ in supra-maximal tests (to reach the VO_2_ peak) or by exhaustive protocols (to achieve maximum O_2_ consumption—VO_2_max—even with an increase in intensity, VO_2_ does not increase) (19). Previous studies have identified the 400-m front crawl as a reliable test to assess the maximum aerobic power and anaerobic contribution (20–22). As the average speed in the 400-m front crawl test is similar to the minimum speed to reach the VO_2_max, the energy profile of this test deserves attention in relation to the possibilities of its use to prescribe training intensities, for example (20). Meta-analysis and systematic reviews are scarce in research concerning observational studies related to sport performance, and specifically swimming (23, 24). Therefore, the purposes of this study were to summarize, analyse, and evaluate results of studies involving 400-m front crawl kinematic, arm-stroke efficiency, coordination, and energetic parameters, tthrough a systematic review with meta-analysis. This meta-analysis can provide to coaches and sports scientists an overview of the 400-m front crawl test.

## Methods

The Preferred Reporting Items for Systematic Reviews and Meta-Analyses (PRISMA) statement check list was used for this meta-analysis, following the established criteria (25).

### Data strategy and sources

The search strategy used in the PubMed, Embase, SPORTSDiscus and Web of Science databases for study collections were: swimmer AND swimming OR “front crawl” OR “middle distance” OR “400 m” AND energy OR kinematic OR coordination OR efficiency. Only complete original articles were selected and they were published between January 1970 and December 2022. The Boolean operators “AND” and “OR” were used for tracing during the searches in the electronic databases.

### Eligibility criteria

The meta-analysis included studies that: (i) performed observational analysis; (ii) analyzed competitive swimmers of both sexes; (iii) used the 400-m crawl test as a protocol. On the other hand, studies were excluded when: (i) participants’ age were equal and less than 12 years old and did not clearly show the age mean; (ii) results were incomplete for standard deviation on the results; (iii) were without information from anthropometric data; (iv) described paralympic swimmers; (v) duplicate from the same authors and sample size.

### Performance outcomes parameters

To facilitate the construction of the meta-analysis, only one main outcome representing each performance parameter was determined in agreement with the research team. Therefore, the performance outcomes extracted from these studies were: (i) kinematics (clean swim speed); (ii) arm-stroke efficiency; (iii) coordination (index of coordination); (iv) energy (peak and maximal oxygen consumption). The swimming speed was chosen to represent the kinematic parameters by representing the performance in the 400-m front crawl test. The extraction of the values (m·s^−1^) for meta-analysis were of the studies that evaluated the clean swim speed either by photogrammetry or by the chronometer. The ηp ((v·0.9)⁄(2π·SR·l)·2⁄(π)*100) in %) was obtained by the paddle wheel method in all the selected studies (3).

The selection of studies regarding coordination parameters were those that presented the results of the IdC (%). All selected studies used the qualitative class model previously proposed (4).

The representative variable of the energy parameters was the VO_2_peak or VO_2_max obtained in the 400-m front crawl test. The methodological protocols for obtaining VO_2_ peak values were by retro-extrapolation and direct method during the test. The first is obtained after the end of the test. The second consists of obtaining the VO_2_ breath-by- breath during the entire test. The VO_2_peak is the highest value considered until the end of the test and the VO_2_max is derived from incremental protocols to exhaustion. Both methods have already been previously reported (20, 21).

### Results extraction and assessing risk of bias

The data were extracted by two independent researchers with experience in systematic review and meta-analysis. In the first phase, the studies were selected and gathered by analyzing the databases described in the search strategy. In the second phase, the exclusion criteria established in the previous extraction were applied. First, duplicates were excluded and then, by reading the abstract and, if necessary, the full paper, which did not include the eligibility criteria. A third researcher was requested when there were divergences in the inclusion and exclusion criteria. All researchers maintained the same pattern in the extraction of meta-analysis data.

In addition, the researchers evaluated the methodological quality of the selected articles. For this, the Downs and Black Quality Assessment Checklist (26) for studies with and without randomization was used. The original version of the scale includes 27 items referring to the classification of the methodological quality of the studies. However, some adaptations were made to better fit the focus of this research: (i) item 27 was not used for evaluating whether the negative findings from the study could be due to chance; (ii) replacement of “patient” by “participant” and “treatment” by “testing”. These changes in the assessment of the quality were also carried out in previous studies (24, 27, 28). The points obtained were totaled (fraction between the total number of points reached and total number of applicable points), followed by conversion into percentages, like previous study (24).

### Data analysis

The kappa index was used to demonstrate the reliability between the researchers in the scoring procedure. The interpretations of the degrees are: (i) very low: 0 < *K* < 0.19, (ii) considerable: 0.20 < *K* < 0.39, (iii) moderate: 0.40 < *K* < 0.59, (iv) substantial: 0.60 < *K* < 0.79, and (v) excellent: 0.80 < *K* < 1.00 (29). For the meta-analysis, the mean and standard deviation of kinematics, arm-stroke efficiency, coordination, and energy parameters obtained from the selection of studies were used. When necessary, a conversion to equal units of measure was performed. A random effect model was developed for the data statistics (30). The heterogenicity, represented by the inconsistency test (*I*^2^), was applied among the selected studies whose results represent: 0%–25% low; 25%–50% moderate; 50%–75% high, over 75%, very high (30). The weight quality of the studies (31) was calculated by the inverse of the square standard error ($1/SE^{2})$ and converted into per cent. The risk of data bias was observed by the forest plot (developed manually in Graph Prism 8.0) and statistic results (*I*^2^, SE and CI95%) were provided by the software Open Meta Analyst (32). Subgroup analyses were performed to minimize the effect of inconsistency. The alpha was set at 5%.

## Results

**Figure 1:**
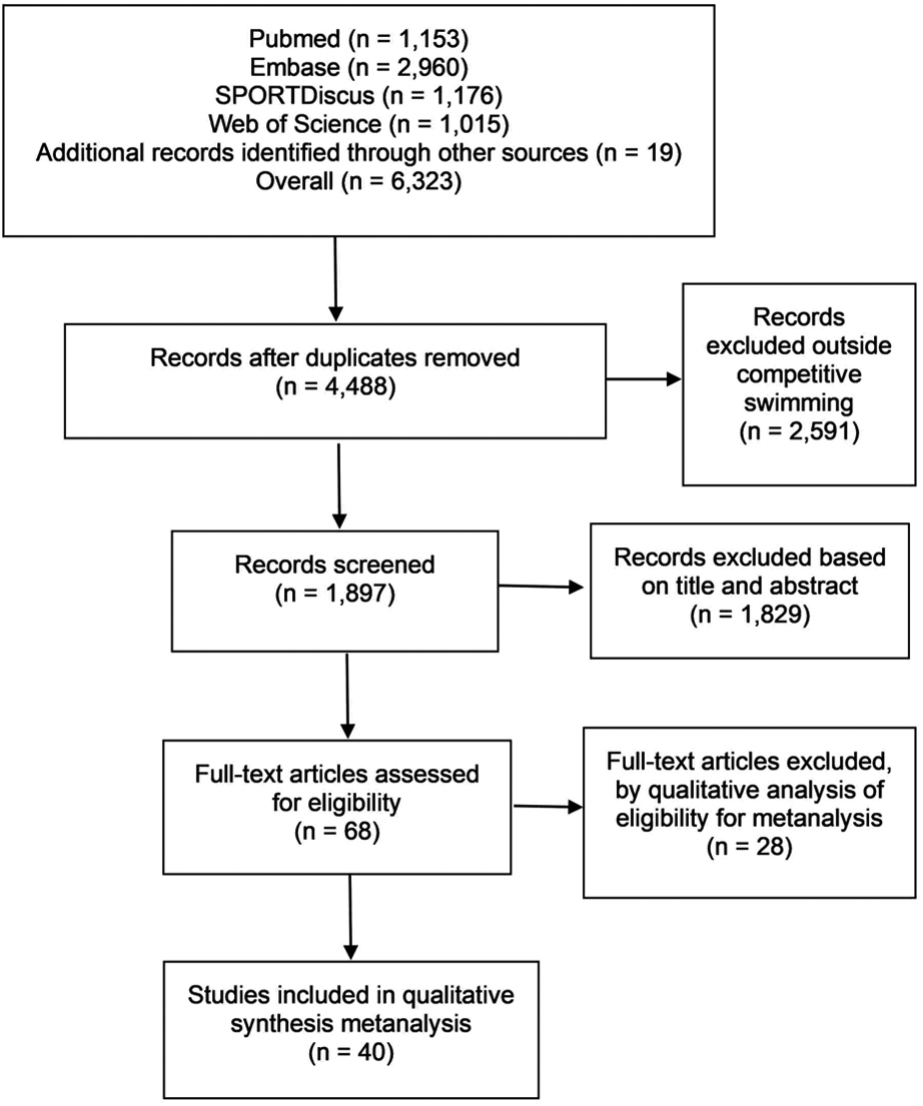
Flowchart summary of the studies selection process.

The research first identified 6,323 studies, of which, after the exclusion of duplicates, 4,488 remained. With the analysis by means of the titles and abstracts, 2,591 studies were excluded (1,897 studies included). Subsequently, 68 studies were identified in the analysis of the complete articles following the eligibility criteria. Afterwards, 28 studies were excluded for the following reasons: (i) results did not correspond to the maximum 400-m front crawl test; (ii) swimmers of very young age; (iii) when it was not possible to identify the described results; (iv) articles that did not provide sample characterisation data (for example: body mass to convert the unit of measurement of oxygen consumption); (v) articles by the same author, but with the same sample characterisation (in this case only one was chosen). Thus, 40 studies completed the final analysis and inclusion criteria for the meta-analysis (Figure 1).

In total, 40 articles were examined extensively because they fitted the previously established inclusion criteria. Of these, 28 studies were classified as moderate and 12 as high quality. No studies were considered low or very high quality. The reliability between both reviewers showed an almost perfect agreement (*k* = 0.85—excellent; *p* ≤ 0.001). Table 1 describes the studies with the quality score, swimmers’ competitive level, sample size, time trial, swimming speed, *ηp*, IdC and VO_2_.

**Table 1:** Studies, quality score, swimmers’ competitive level, sample size, time trial, swimming speed, arm-stroke propelling efficiency (*np*), index of coordination (IdC) and oxygen consumption (VO_2_).

Studies	Score (%)	Swimmers (competitive level)	Sample	Time trial (s)	Swimming speed (m·s^−1^)	*Ηp* (%)	IdC (%)	VO_2_ (ml·kg^−1^·min^−1^)
Arsoniadis, Bogdanis (13)	53.8	Regional and national swimmers	♂*N* = 12	-	1.28 ± 0.10 (experience group)	34.0 ± 4.0 (experience group)	-	44.0 ± 6.0 (experience group)
Barbosa, de Jesus (14)	42.3	Sub elite	♂*N* = 22	254.9 ± 20.4	1.28 ± 0.40	30.0 ± 5.0	−13.0 ± 7.0	-
Bassan, César (33)	34.6	Competitive	♂*N* = 15		1.35 ± 0.15	-	-	-
Bentley, Roels (34)	57.7	Elite	♂♀*N* = 8	265.0 ± 15.0	1.30 ± 0.06	-	-	51.2 ± 5.8
Campos, Kalva (35)	34.6	Competitive	*N* = 9 (♂*N* = 6 ♀*N* = 3)	-	1.34 ± 0.13	-	-	62.3 ± 13.7
Chatard, Collomp (36)	42.3	Competitive	♂*N* = 9		♂1.49 ± 0.07	-	-	-
Chatard, Chollet (37)	50.0	Triathletes	♂*N* = 8	297.25 ± 7.24	1.35 ± 0.01	-	-	64.8 ± 2.9
Chollet, Hue (38)	42.3	Competitive	♂*N* = 6	-	1.34 ± 0.02 (no drafting)	-	-	65.2 ± 1.2 (no drafting)
Correia, Feitosa (20)	42.3	Competitive	♂*N* = 14	316.36 ± 0.21	1.26 ± 0.11	-	-	68.1 ± 9.7
Dalamitros, Zafeiridis (39)	53.8	Competitive	(control group; *N* = 8)	control group 324.13 ± 16.97	control group 1.25 ± 0.01	-	-	-
Dekerle, Sidney (40)	53.8	Well trained	♂*N* = 8 ♀*N* = 2	-	♂♀1.42 ± 0.10 (second part)	-	-	-
Deminice, Gabarra (41)	42.3	Competitive	♂*N* = 9 ♀*N* = 5	-	1.38 ± 0.09	-	-	-
Fernandes, Sousa (42)	42.3	Long distance	♂*N* = 17	-	1.29 ± 0.16	-	-	-
Franken, Figueiredo (43)	42.3	Competitive	♂*N* = 11	-	1.45 ± 0.08	-	-	-
Funai, Matsunami (44)	42.3	Well-trained national-level	♂*N* = 7	264.98 ± 8.94	1.46 ± 0.05	-	-	-
Gay, López-Contreras (12)	53.8	Triathletes and open water	♂*N* = 33	-	1.17 ± 0.16 swim	40 ± 6.2	-	-
Greco, Pelarigo (45)	42.3	Trained	♂*N* = 22; ♀*N* = 14	-	Overall = 1.38 ± 0.09; ♂ = 1.32 ± 0.10; ♀ = 1.21 ± 0.10	-	-	-
Jurimae, Haljaste (46)	34.6	Competitives Youngs	♂*N* = 29	378.3 ± 53.5	1.05 ± 0.14	-	-	62.0 ± 19.4
Kalva-Filho, Campos (22)	42.3	Competitive	♂*N* = 12	-	1.40 ± 0.10	-	-	63.2 ± 13.7
Laffite, Vilas-Boas (9)	38.5	Trained	♂*N* = 7	256 ± 7	1.56 ± 0.04	-	-	67.2 ± 5.6
McCabe and Sanders (47)	46.2	Sprint and specialists’ middle distance	♂*N* = 15 (*N* = 7 sprints, *N* = 8 middle distance)	242.59	Overall: 1.45 ± 0.05	-	-	-
Mezzaroba, Papoti (48)	42.3	Trained	*N* = 33 (♂*N* = 17 and ♀*N* = 16)	-	♂ = 1.18 ± 0.19; ♀ = 1.01 ± 0.10; Y: 1.09 ± 0.14	-	-	-
Obert, Falgairette (49)	42.3	Regional	*N* = 13 (♂*N* = 12 and ♀*N* = 8)	352 ± 52	1.06 ± 0.10 (*N* = 13)	-	-	-
Oliveira, Caputo (50)	42.3	Trained	♂*N* = 13	-	1.29 ± 0.05	-	-	-
Papoti, Silva (51)	50.0	National	*N* = 21 (♂*N* = 5 and ♀*N* = 9)	-	Y: 1.24 ± 0.09	-	-	-
Petibois and Deleris (52)	46.2	Competitive	♂*N* = 7	255.9 ± 6.8	1.54 ± 0.06	-	-	
Ribeiro, Cadavid (6)	42.3	Competitive	♂*N* = 15	296 ± 68	1.44 ± 0.05	-	-	56.0 ± 6.0
Rodríguez and Mader (53)	42.3	Young	♂♀*N* = 10	-	♂1.44 ± 0.01; ♀1.36 ± 0.04	-	-	63.2 ± 6.0
Rodriguez (54)	65.4	National Spanish	serie A *N* = 15 (♂*N* = 10 and ♀*N* = 5; serie B *N* = 33 (♀*N* = 22 and ♂*N* = 11)	serie *A* = 265.60 ♂; 280.30 ♀; serie *B* = 272.11s ♂, 294.33s ♀(time trial based on high speed reported in study)	serie A: ♂1.51 ± 0.07, ♀1.43 ± 0.09;	-	-	62.2 ± 10.0
Samson, Monnet (55)	34.6	Regional and national	*N* = 17 (♂*N* = 9 and ♀*N* = 8)	-	1.51 ± 0.08 (*N* = 17)	-	-	-
Schnitzler, Ernwein (56)	46.2	National, international and recreational	*N* = 34 (recreational: ♂*N* = 8 and ♀*N* = 9; expert ♂*N* = 8 and ♀*N* = 9; overall ♀ = 18	-	Recreational: ♂1.21 ± 0.06, ♀ = 1.02 ± 0.07; Expert: ♂1.45 ± 0.05, ♀ = 1.35 ± 0.07; Overall♀ = 1.18 ± 0.07	-	-	-
Schnitzler, Seifert and Chollet (15)	50.0	Competitive	♂*N* = 16 (G1 *N* = 8 expert swimmers; G2 *N* = 8 students with lower performances.	G1 = 267.6 ± 9.9 G2 = 344 ± 14.9	G1: V50 = 1.49 ± 0.08; G2: 1.18 ± 0.11; ♂1.33 ± 0.05	-	−12.5 ± 3.7	-
Schnitzler, Seifert (16)	42.3	French highly trained	♂*N* = 9	280 ± 5.6	Trial 2: 1.39 ± 0.1	-	−13.8 ± 4	-
Strzala, Tyka (18)	34.6	Competitive	*N* = 26		1.42 ± 0.07		−5.9 ± 6.55	
Tsalis, Toubekis (57)	42.3	Different years	*N* = 11 young; *N* = 7 adults	*Y* = 332.3 ± 23; *S* = 315.2 ± 14.6	*Y* = 1.16 ± 0.11); *S* = 1.21 ± 0.08	-	-	-
Wakayoshi, Ikuta (58)	38.5	College	♂*N* = 9		1.54 ± 0.07	-	-	57.8 ± 6.6
Zacca, Fernandes (59)	30.8	Competitive	*N* = 10 (♂*N* = 7 and ♀*N* = 3)	278 ± 16 0.9	1.44 ± 0.08	-	-	64.5 ± 8.6
Zacca, Azevedo (60)	57.7	Young	*N* = 24 (♂*N* = 10, ♀*N* = 14)	315 ± 22 (E4)	1.21 ± 0.08	-	-	49.9 ± 5.2
Zacca, Azevedo (21)	57.7	National level	*N* = 11 (♂*N* = 6, ♀*N* = 5)	311 ± 17	1.28 ± 0.07; Y: 1.21 ± 0.09	-	-	54.4 ± 6.6
Zacca, Toubekis (61)	57.7	Young	*N* = 15 (♂*N* = 6, ♀*N* = 9)	358 ± 21 (Pós)	1.11 ± 0.07; Y: 1.29 ± 0.07	-	−8.5 ± 4.1	42.3 ± 6.6; Y: 45.4 ± 5.7

♂, male; ♀, female; S, senior swimmers; Y, young swimmers.

### Kinematic, arm-stroke efficiency, coordination, and energetic parameters

**Figure 2:**
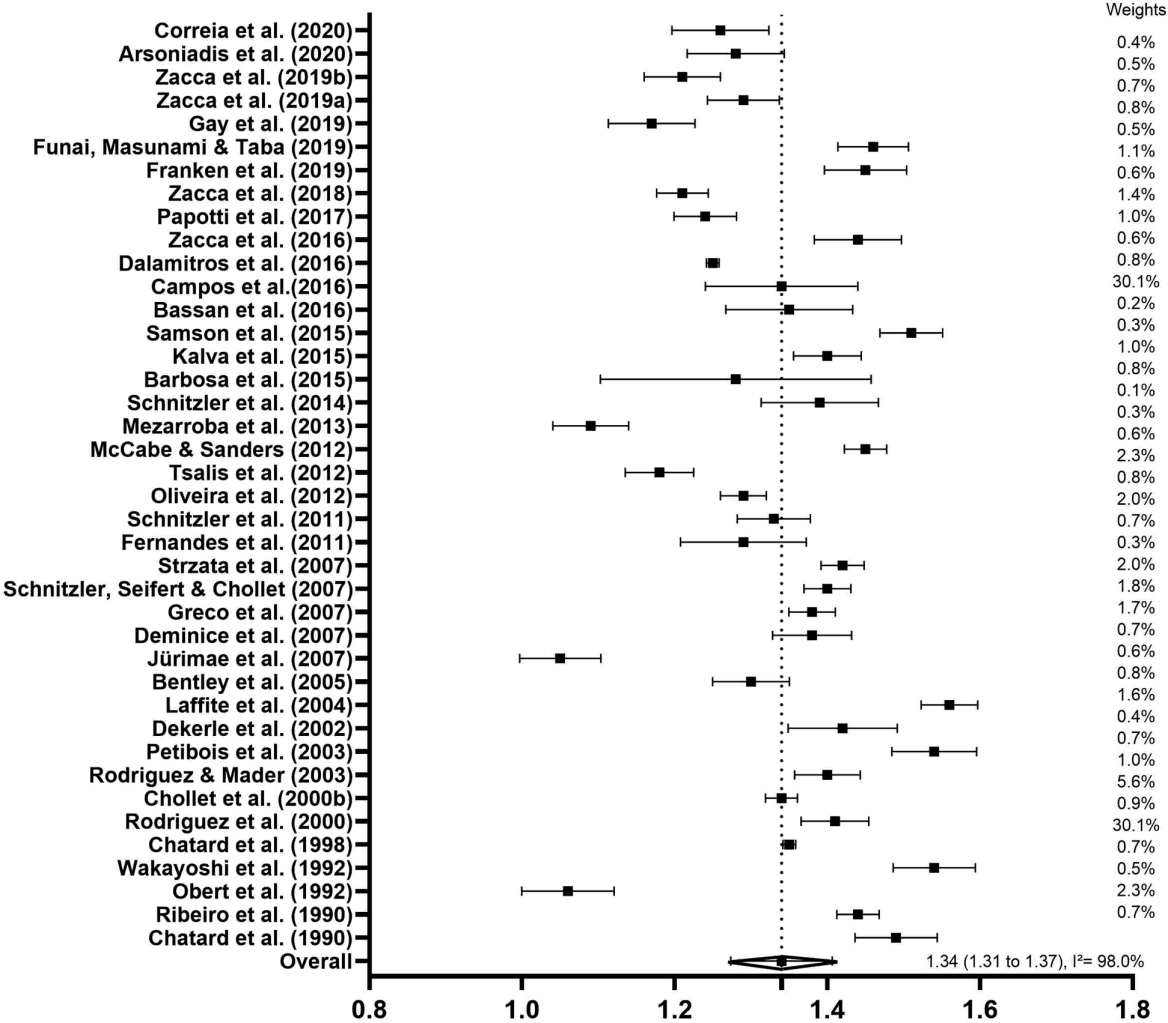
Forest plots derived from continuous random-effects models depicting the kinematics parameters represented by clean swim speed (m·s^−1^) in 400-m front crawl test.

Due to the large number of studies describing swimming speed, it was possible to carry out subgroup analyses to verify the mean swimming speed responses, as well as the variability of the studies divided into categories. Figure 2 shows the forest plot with the inclusion of the 40 overall studies (*n* = 611) representing the clean swim speed in the 400-m front crawl. The mean swimming speed was considered moderate (1.34 m·s^−1^ and SE = 0.02; 95% CI: 1.31 to 1.37 m·s^−1^). The heterogeneity was considered high (*I*^2^ = 98%, *p* < 0.01), which shows a high variability across the studies.

**Figure 3:**
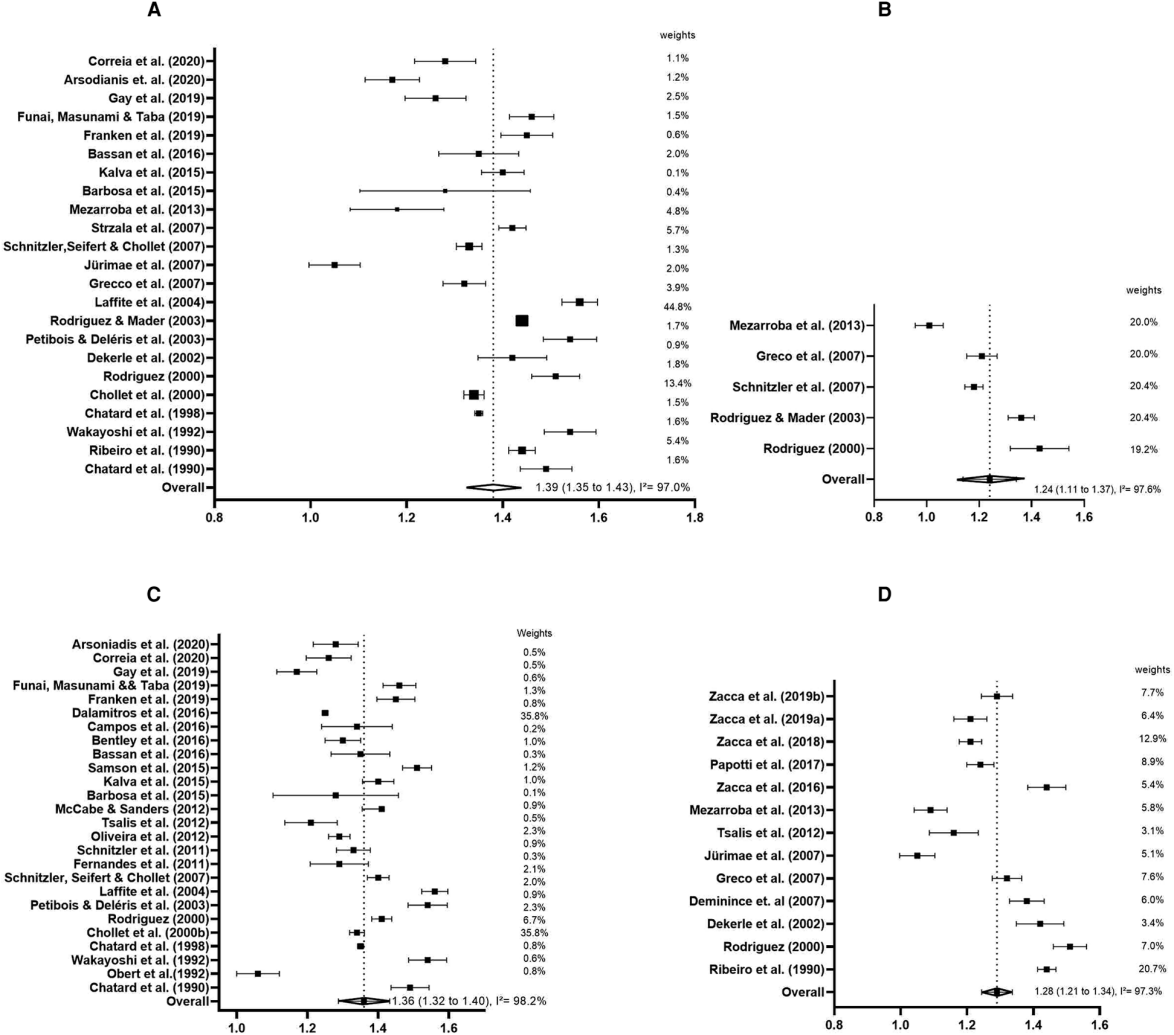
Forest plots and subgroup analyses (gender category = **A**: male and **B**: female, respectively; divided by age category = **C**: senior and **D**: junior swimmers derived from continuous random effects models depicting the swimming speed (m·s^−1^) in 400-m front crawl test.

Next, an analysis by subgroup of the gender (male and female) for swimming speed (Figure 3A,B) was developed. So, the meta-analyses were performed with 22 studies for male swimmers (*n* = 244) and five studies for female (*n* = 70). The mean swimming speed of male swimmers was 1.39 m·s^−1^ (SE = 0.02; 95% CI: 1.35 to 1.43 m·s^−1^), higher when compared to the mean swimming speed of female swimmers: 1.24 m·s^−1^ (SE = 0.07; 95% CI: 1.11 to 1.37 m·s^−1^). The heterogeneity was considered high for both genders (male: *I*^2^ = 97.0%, *p* < 0.01; female: *I*^2^ = 97.6%, *p* < 0.01), while the high variability between studies in each category remained.

Subsequently, the analysis by subgroup was developed by senior (over 18 years old) and junior (under 18 years old) categories for swimming speed (Figures 3C,D, respectively). The criteria used by the junior and senior categories was the age group based on the previous study (62). Twenty-six studies were included in the senior category (*n* = 205) and 14 studies in the junior category (*n* = 242). The senior category showed a higher mean swimming speed of 1.36 m·s^−1^ (SE = 0.03; 95% CI: 1.32 to 1.40 m·s^−1^) compared to the junior category swimming speed, which had a mean of 1.28 m·s^−1^ (SE = 0.03; 95%CI: 1.21 to 1.34 m·s^−1^). The heterogeneity remained large for both categories (senior category: *I*^2^ = 98.2%, *p* < 0.01; junior category: *I*^2^ = 97.3%, *p* < 0.01), demonstrating a large variability of studies in both categories.

**Figure 4:**
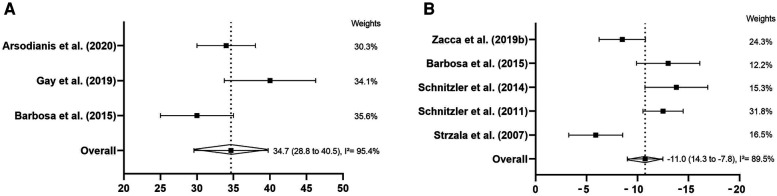
Forests plots derived from continuous random-effects models depicting the efficiency (**A**: % ηp = arm-stroke propelling efficiency) and coordination (**B**: % IdC = index of coordination) parameters in 400-m front crawl test.

Figure 4A,B shows the *ηp* and the IdC's meta-analysis (respectively). There was a total of three studies with *ηp* (*n* = 67) and five with IdC (*n* = 73). The *ηp* was considered high, with mean of 35% (SE = 2.97; 95% CI: 28.8% to 40.5%). The heterogeneity was considered high (*I*^2^ = 95.4%, *p* < 0.001) due to the high variability of the studies. The IdC showed that swimmers have adopted the catch-up coordination model, with an IdC mean of −11% (SE = 1.65; 95% CI: −14.3% to −7.8%). The heterogeneity was high (*I*^2^ = 89.5%, *p* < 0.001 for IdC).

**Figure 5:**
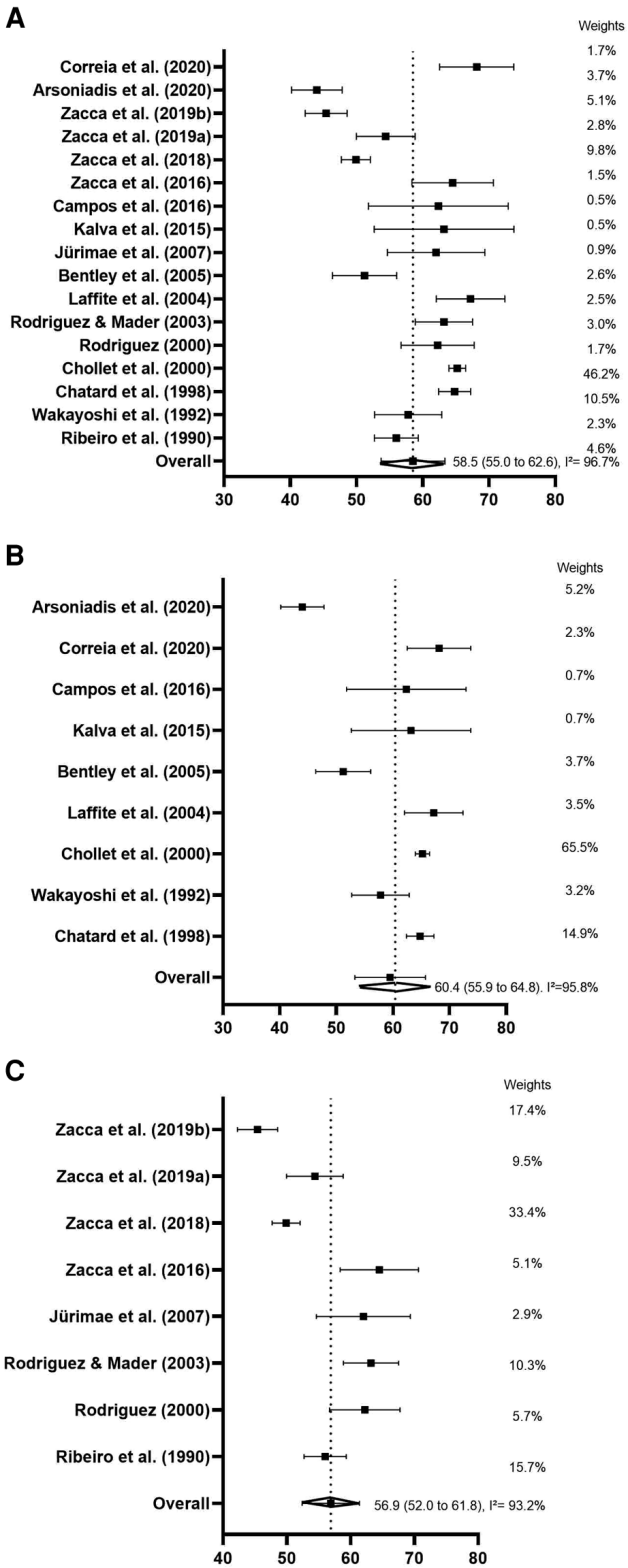
Forest plots and subgroups analysis (**A**: mean overall VO_2_; **B**: senior category and **C**: junior category, respectively) derived from continuous random-effects models depicting the energy parameters (VO_2_: oxygen consumption expressed in ml·kg^−1^·min^−1^).

Figure 5A shows 17 studies (*n* = 211) that were selected in the VO_2_ meta-analysis. The overall mean of VO_2_ was high, corresponding to 58.5 ml·kg^−1^·min^−1^ (SE = 2.06). The heterogeneity was considered high (*I*^2^ = 96.7%, *p* < 0.01) due to the variability of studies. Figure 5B,C shows the analysis of subgroups by age category (senior and junior, respectively). In the senior category, nine studies were included (*n* = 82), and the VO_2_ mean was higher, 60.4 ml·kg^−1^·min^−1^ (SE = 2.06; 95% CI: 54.7 to 64.2 ml·kg^−1^·min^−1^) when compared with the junior category (eight studies; *n* = 129), where VO_2_ average was 56.9 ml·kg^−1^·min^−1^ (SE = 2.5; 95% CI: 52.0 to 61.8 ml·kg^−1^·min^−1^). The heterogeneity remained high for both subgroups (senior: *I*^2^ = 96.2%, *p* < 0.01; junior: *I*^2^ = 93.3%, *p* < 0.01).

## Discussion and implications

The purposes of this study were to summarize, analyse, and evaluate results of studies involving 400-m front crawl kinematic, arm-stroke efficiency, coordination, and energetic parameters, through a systematic review with meta-analysis. This study tried to demonstrate an overview of the performance parameters of the 400-m front crawl test. Swimming speed, *ηp*, IdC, and VO_2_ results were analyzed. In an overall analysis of the results, independent of gender, the results indicated that the 400-m front crawl mean speed was moderate (1.34 m·s^−1^), with large ηp (35%), IdC was in the catch-up coordination model (−11%), and swimmers reached high VO_2_ values (59 ml·kg^−1^·min^−1^). However, when compared with only the best results found, these mean results are below of those reported previously: speed 1.56 m·s^−1^ of seven trained male swimmers (9); ηp: 40.0% of 33 open water and triathletes, male and female swimmers (12); IdC: −5.9% in 26 competitive male swimmers (18); VO_2_ = 68.1 ml·kg^−1^·min^−1^ in 14 competitive male swimmers (20). This comparison is limited by the gender, but the cited values were the highest of each parameter analyzed in this meta-analysis.

The assessment of bias risk from the selected studies was performed with the checklist previously reported (26). The score results (converted to percentage for better understanding of the parts by the whole) of study similar (27) to the present one were approximately 45%. Studies related to swimming performance (27, 63) obtained from 35% to 41%. This can be explained by the fact that most studies were not as rigorous in terms of delineation (e.g., clinical and randomized trials). Implications of group analysis, intervention and blinding of participants, for example, common to these types of trials mentioned, had to be considered null within the scale score. In addition, some items had to be adapted to better suit observational studies (24). Therefore, the meta-analysis pointed to a lack of studies with better methodological quality. It is clear that randomized, clinical, and longitudinal studies can provide better cause and effect responses of parameters that are responsible for the performance of a given event in any type of competitive sport, as previously pointed out (27, 64). Research in the area of sport performance, and swimming, has the difficulties of recruited high sample sizes, making studies with more robust qualities difficult (63). On the other hand, it is understood that such methodologies are often not in accordance with the aims of the studies that were analyzed in the present meta-analysis. Future researches should use more directed to the area scales of evaluation of the quality of studies and that encompass the reality of studies related to sports performance, specifically in competitive swimming.

Over the decades, the 400-m front crawl test has been investigated to help swimmers and coaches improve performance in competitive events (20, 59). It is already known that swimming speed is the parameter that best represents a swimmer's performance (46). The faster the swimmer is (and the more able to sustain high speed), the shorter the time to complete the desired distance, which is reflected in the best performance. Thus, the swimming speed was chosen to represent the kinematic parameters. The average result of the swimming speed (male and female together) reached ≈75.7% of the average speed of the world records of the 400-m freestyle (this speed was calculated as the average of the speeds, with start and turn contributions, of the world records of the 400-m freestyle in 50 and 25-m swimming pools for male and female, retrieved from www.fina.org). This result could be explained by: (i) lower competitive level swimmers are more available to assess in laboratories; (ii) test conditions that do not provide a competitive environment; (iii) the open turns and lacking underwater phase after turns when using a snorkel with valve for respiratory gas analysis. Therefore, a subgroup analysis was conducted to identify whether any factor would reduce heterogeneity and raise mean swimming speed values. Following the eligibility criteria, it was possible to divide the studies into two subgroups: (i) gender (male and female); (ii) age-group categories (senior and junior). No analysis was able to reduce heterogeneity and there was still great variability among the studies (however, as expected, male swimmers reached higher speeds than female swimmers).

Male swimmers can achieve higher speeds than female swimmers in competitive swimming events (65). This is explained by the fact that male swimmers have larger body segments and increased muscle strength. In addition, male swimmers can perform higher SR and SL, to overcome hydrodynamic drag (66). About the subgroup of competitive categories, senior swimmers reached higher swimming speeds (even higher than the mean of all general studies) compared to junior swimmers. This can be explained by the training and competitive experience over the years in the sport (67). In one of the studies that showed the highest speed in the 400-m was (9), the swimmers selected were part of the French national team and obtained high performance in the test (≈256 s). The study that reported the poorest test (≈378 s) (46) assessed male junior swimmers. Furthermore, only in five studies (9, 52, 54, 55, 58) swimmers reached swimming speeds over 1.50 m·s^−1^; however, the swimmers of these cited studies were of optimal technical level. Therefore, more studies are needed to include high-performance swimmers and separated groups to perform analysis of performance (e.g., technical level, genders, and competitive categories), and to verify which performance parameters reflect the actual performance.

It was not possible to carry out an analysis of subgroups in *ηp*, due to the small number of studies. The mean result (34.6%) shows that expert swimmers, in the 400-m front crawl test, keep their *ηp* high and close to the values previously referenced of ≈40% (3). In addition, one of the studies included (12) reported the same *ηp* with the use of a swimsuit by the swimmers and concluded that the high *ηp* obtained at this event is due to the large SL. Only five selected studies evaluated coordination parameters with the IdC. The mean result (−11.0%) indicated the catch-up model, previously described (4), as that performed by the swimmers. It is already known that the different coordination models do not represent the best or the worst performance, but can be an adaptation that swimmers acquire to overcome hydrodynamic drag oscillations, mainly when speed is increased. However, two studies obtained higher IdC compared to the mean. Previous study (18) reported IdC = 6%. However, the same study reported a mean speed of 1.42 m·s^−1^, which does not represent the best test performance among the studies included in the meta-analysis. Possibly, the high IdC value is related to the increased SR. Regarding both, *ηp* and IdC, more studies that verify this parameter are needed for a better understanding of the results.

VO_2_ was the chosen energy parameter. Seventeen studies were selected and the VO_2_ mean was 58.8 ml·kg^−1^·min^−1^, which represents great aerobic power. The division of the subgroup indicated that the VO_2_ of senior swimmers was like that of junior swimmers. So, the age difference has no influence on the values achieved. However, one should consider the swimming speed related to the VO_2_ values. In the study (9) that reported the highest swimming speed, the swimmers reached up to 67.0 ml·kg^−1^·min^−1^ at 1.56 m·s^−1^. Study with junior male competitive swimmers (46) reported 62.0 ml·kg^−1^·min^−1^ at 1.05 m·s^−1^, with high VO_2_, but low swimming speed. High VO_2_ values could be related to test duration (from ≈ 242 s to ≈ 378 s). Improved buffering conditions (68), recruitment of fast-contractile muscle fibres, and aerobic power development (69) are some examples of the high VO_2_ values’ importance in swimming performance. In addition, high swimming speed and high VO_2_ are targets to be reached in training for middle- and long-distance specialist swimmers, mainly to develop improved metabolic power. Therefore, future studies should direct a better analysis to the speed reached in the permanence of VO_2_ next to maximum.

The results indicated that the 400-m front crawl test presented sub-maximum speeds, required a high arm-stroke efficiency, is performed under catch-up coordination model, and reached high energy demand. However, arm-stroke efficiency and aerobic power do not support the best 400-m front crawl test performance (here represented by the swimming speed). The analysis of subgroups (to identify which factors influence the heterogeneity of the studies), showed that swimmers can reach higher speeds and, consequently, by swimming technical level and specialty, they can decrease the test time and increase performance. Thus, we understand that the swimming speed seems to be the more influential in changing other parameters (efficiency, coordination, energy) in the 400-m front crawl performance.

However, many researchers of sport sciences get stuck in the sample size requirements to have robust and demanding inferential statistics. In this context, the individuals selected to participate in the research are swimmers with easy accessibility, so it is possible to reach the required sample number (for example: university, regional, and national swimmers of age categories, master swimmers). Those of international and elite level are more difficult to assess due to competitive and training schedules. In this regard, the present meta-analysis also pointed out that these level differences determine the high heterogeneity found in the performance parameters. Moreover, the results demonstrated the 400-m front crawl performance parameters’ characteristics. Thus, this meta-analysis results can help coaches and researchers to monitor and improve performance, and develop further research in the field of swimming. On the other hand, we can indicate the lack of appropriate evaluation scales for studies with the characteristics of those that fit the eligibility criteria of the present study. Such studies respond very well to the proposed objectives but end up having moderate evaluation in recognized scales.

## Data Availability

The original contributions presented in the study are included in the article/Supplementary Material, further inquiries can be directed to the corresponding author.
